# Effect of the intestinal microbiota on T_H_17 cell-derived cytokines in the context of intestinal inflammation

**DOI:** 10.1007/s00281-026-01070-3

**Published:** 2026-04-02

**Authors:** Justus Neuendorff, Samuel Huber, Penelope Pelczar

**Affiliations:** 1 Department of Medicine, University Medical Center Hamburg-Eppendorf, Hamburg, Germany; 2 Hamburg Center for Translational Immunology (HCTI), Hamburg, Germany

**Keywords:** Inflammatory bowel disease, Intestinal microbiota, Mucosal immunity, T_H_17 cells

## Abstract

Inflammatory bowel disease (IBD) is a chronic and relapsing inflammatory condition of the gastrointestinal tract (GI tract). Despite extensive research, its exact pathogenesis remains elusive. However, multiple factors are thought to be involved in the onset and progression of IBD. Aside from genetic risk factors, microbial dysbiosis, environmental cues, defects in the epithelial barrier, as well as a dysregulated intestinal immune response, are critical players driving a vicious cycle, which ultimately results in chronic disease. An orchestrated interaction between the intestinal microbiota and the immune response, especially T_H_17 cells, is critical for maintaining and re-establishing intestinal homeostasis. Nonetheless, a misguided interaction contributes to the pathogenesis of IBD. Indeed, depending on the microenvironment, intestinal T_H_17 cells possess dual properties. Either they act to control the inflammatory response, or they acquire pro-inflammatory features promoting the development of intestinal pathology. This context-dependent phenotype of T_H_17 cells has recently been associated with the microbiota composition, which shapes the inflammatory milieu of the gut. To establish precision immunomodulation as a therapeutic strategy for patients with IBD, it is critical to understand how intestinal microorganisms are involved in actively directing the dichotomous nature of T_H_17 cells and their cytokine products.

## T_H_17 cells are part of the intestinal adaptive immune response

CD4^+^ T helper cells (T_H_ cells) are a heterogeneous population of T cells and an essential component of the adaptive immune response. They orchestrate various defense mechanisms to protect the body against invading pathogens. For several decades, the T_H_1/ T_H_2 paradigm described how different CD4^+^ T_H_ cell subsets with distinct cytokine profiles would control the body’s immune response towards a specific agent [1, 2]. This dualistic model was challenged by the discovery of an additional subset of interleukin-17 A (IL-17 A) producing CD4^+^ T_H_ cells, now known as T_H_17 cells, in murine models of autoimmune inflammation [3–6]. T_H_17 cells primarily control the body’s immune response towards extracellular bacteria and fungi, especially at barrier sites. However, T_H_17 cells possess a dichotomous nature since they are also implicated in the development of tissue inflammation and autoimmune diseases [7].

## Cytokine profile of T_H_17 cells

The retinoid orphan receptor gamma t (RORγt) was identified as the master transcription factor to regulate T_H_17 cell lineage commitment [8]. Besides IL-17 A, T_H_17 cells can secrete a variety of different cytokines including IL-17 F, IL-21, IL-22 and granulocyte-macrophage colony-stimulating factor (GM-CSF). To a lesser extent, T_H_17 cells have also been shown to produce tumor necrosis factor-α (TNFα), as well as IL-6 [3, 9, 10]. In addition, T_H_17 cells are characterized by their expression of the chemokine receptor type 6 (CCR6), mediating their recruitment to the inflammatory site [11]. Moreover, T_H_17 cells can co-express other cytokines, which reflects on their functional heterogenicity. Double positive (DP) IL-17 A^+^INFγ^+^ CD4^+^ T_H_ cells are associated with intestinal pathology and can be found in inflammatory lesions of patients with IBD [12–14]. In the gut, the co-expression of IL-10 along with IL-17 A is associated to an anti-inflammatory T_H_17 phenotype [7, 15–17].

### IL-17 A and IL-22, two important T_H_17 cell derived cytokines

Among the variety of cytokines secreted by T_H_17 cells, IL-17 A and IL-22 have emerged as central mediators in mucosal immunity. IL-17 A is known to be the signature cytokine of T_H_17 cells. Within the IL-17 family of cytokines, consisting of six members (IL-17 A-F) characteristic structural motifs are shared. Among them solely IL-17 A and IL-17 F are produced by T_H_ cells. However, their production is not an exclusive feature of CD4^+^ T_H_ cells. Both IL-17 A and IL-17 F bind to their corresponding transmembrane receptor, which is composed of two subunits (IL-17RA/IL-17RC). Various tissues, such as epithelial and mesenchymal cells, as well as leukocytes, express this heterodimeric receptor on their cellular surface [18].

IL-22 is part of the IL-10 family of cytokines. Its heterodimeric transmembrane receptor (IL-10Rb/IL-22Ra1) is expressed in various organs, including the intestine, the skin and the liver. As the IL-10Rb is ubiquitously expressed throughout the whole body, IL-22 signaling is restricted by the expression of the IL-22ra1 subunit. The latter is limited to cells from the non-hematopoietic origin, such as intestinal epithelial cells (IECs). Thus, unlike several other cytokines, IL-22 does not directly interfere with immune cells [19]. In the intestine, several CD4^+^ T_H_ cell subsets can produce IL-22. Whereas in humans especially T_H_1 cells co-produce IL-22, in mice T_H_17 cells are the main producers of IL-22. T_H_22 cells are CD4^+^ T_H_ cells that are characterized by the expression of IL-22 in the absence of another signature cytokine, e.g. IL-17 A, IFNg, IL-4, and IL-9 [20]. In addition to CD4^+^ T_H_ cells, also innate immune cells such as γδ T cells, NKT cells as well as innate lymphoid cells (ILCs) contribute to the IL-22 production [21, 22]. Depending on the respective tissue and microenvironment, non-conventional cellular sources of IL-22 were identified. For instance, in intestinal inflammation, production of IL-22 has been attributed to colonic neutrophils [23].

**Table 1:** Effects of IL-17 and IL-22 at the mucosal interface of the intestine

Cytokine	Effect	Mechanism	Reference
IL-17	Regulation of microbiota composition	Production of AMPs	[24]
		Production of epithelial H_2_O_2_	[24]
		Promotion of IgA transcytosis	[24]
	Epithelial barrier maintenance	Formation of Tight Junctions	[25]
	Immune cell recruitment	Promotion of neutrophil recruitment	[26]
IL-22	Epithelial regeneration	Proliferation of ISCs	[27]
		Promotion of wound healing and tissue repair	[28]
	Epithelial barrier maintenance	Production of mucus	[29]
		Formation of Tight Junctions	[19]
	Regulation of microbiota composition	Production of AMPs	[30]
		Production of acute-phase proteins	[31]
		Induction of epithelial fucosylation	[19]

## The intestinal microbiota and mucosal immune tolerance

The gastrointestinal tract of mammalians is colonized by a multitude of different microorganisms, collectively referred to as the intestinal microbiota [32]. This microflora is highly diverse, including bacteria, viruses, and fungi, most of which reside in the colon. Among these, the ratio of bacterial to human cells in the adult body was estimated at 1.1 [33]. The constant presence of foreign antigens presents a unique challenge to the intestinal immune system, which must carefully balance responsiveness and restraint [34]. This equilibrium is achieved through a complex crosstalk between the microbiota and the immune compartment, enabling the host to maintain tolerance while simultaneously controlling bacterial organisms at the mucosal surface.

The intestinal microbiota comprises various types of microorganisms, each with differing potential to cause enteric pathology. Commensal microorganisms colonize the outer and inner surfaces of the human body. They exist in a symbiotic mutualism with their host and typically do not trigger a pro-inflammatory immune response. In the intestine, they carry out several beneficial functions since they facilitate the breakdown of nutritional components and prevent the colonization of pathogenic bacteria [35, 36]. Pathobionts have the potential to promote intestinal pathology under certain conditions. Accumulating evidence strengthens their role in perpetuating intestinal inflammation in IBD, as they can trigger an immune response in a genetically susceptible host. Lastly, classical pathogens possess the ability to cause disease in a healthy individual [37].

The sustained exposure to microbial antigens at the vast mucosal interface of the gut underscores the importance of local immune defense mechanisms. One principle of mucosal defense is to minimize the contact between the bacteria and IECs to restrict an unintentional and excessive immune response. In addition, the proper regulation of tight junctions prevents intestinal bacteria from breaching the epithelial barrier. The gut epithelium has a high turnover rate, constantly renewing to repair potential damage. Second, the bacterial growth is limited by the secretion of AMPs and immunoglobulins (Igs), which shape the composition of intestinal microorganisms [24, 29]. T_H_17 cell-derived cytokines are essential for orchestrating these defense mechanisms. Lastly, intestinal homeostasis involves the tight regulation of balancing the ratio between T_H_17 cells and regulatory T cells (T_reg_).

Amongst the CD4^+^ T_H_ cells, T_reg_ cells are central to establish peripheral tolerance. They actively control the immune response and counteract the development of chronic inflammation [38]. The balance between T_H_17 cells and T_reg_ cells is often referred to as a dynamic counterplay: T_H_17 cells express comparably low amounts of effector cytokines, enabling protection towards luminal microorganisms. In parallel, T_reg_ cells prevent overreaction in response to the constant presence of microbial antigens. Besides, inducible CD4^+^Foxp3^−^ Type 1 regulatory T (Tr1) cells and IL-10 producing T_H_17 cells play central roles in establishing immune regulation, particularly in mucosal tissues [15, 39]. In addition, a Kruppel-like factor 2^+^ (KLF2^+^) Foxp3^−^ subpopulation of Tr1 cells has been shown to protect against commensal microorganisms that do not strongly induce conventional T_reg_ response, thereby contributing to intestinal homeostasis [40]. Often in patients with IBD, the balance between protective and regulatory response in the gut mucosa is disrupted, leading to a breakdown of the intestinal barrier [34]. The leaky epithelium allows an increased microbial translocation, initiating a vicious cycle in which pro-inflammatory cytokines further impair barrier function and exacerbate inflammation. Several genetic risk factors associated with IBD affect the integrity of the mucosal barrier [41]. Intestinal inflammation is accompanied by major changes concerning the composition of the microbiota and patients with IBD feature a reduced microbial diversity but increase variability. Distinct bacterial strains expand in enteric pathology [42]. Generally, it is thought that the combination of genetic risk factors and environmental cues impairs intestinal homeostasis and drives the breakdown of mucosal defense mechanisms and an excessive activation of the intestinal immune system [34].

## Dual effects of T_H_17 cell-derived cytokines in intestinal homeostasis and inflammation

T_H_17 cell-derived cytokines, especially IL-17 A and IL-22, are indispensable to maintain intestinal homeostasis. They mediate both barrier-protective and immune-regulatory functions (Table 1). In intestinal homeostasis, T_H_17 cells are predominantly found in the lamina propria (LP) of the ileum. There, they reside in close contact to the intestinal microbiota [43]. Interactions between these two compartments are known to be bidirectional.

On the one hand, T_H_17 cell-derived cytokines can impact the microbial composition of the intestine and prevent dysbiosis [24, 44, 45]. To control luminal microorganisms, IL-17 A induces the expression of antimicrobial peptides (AMPs), such as α-defensins and initiates the production of hydrogen peroxide (H_2_O_2_) in IECs. Furthermore, IL-17R signaling in IECs is essential for the expression of the polymeric immunoglobulin receptor (pIgR), which enables the transcytosis of secretory IgA [24]. In the lumen, IgA restricts bacterial expansion, neutralizes bacteria-derived toxins and thereby helps to prevent dysbiosis [46]. Additionally, IL-17 A contributes to regulate the permeability of the intestinal barrier [25]. In response to adherent bacteria IL-17 A has been shown to regulated neutrophil recruitment to the small intestine (SI) LP, inhibiting bacterial overgrowth [26].

In parallel, IL-22 contributes to the protection of the epithelial barrier by regulating the expression of *Muc2* in a STAT3 dependent manner [29]. IL-22 promotes the formation of intestinal tight junctions, stimulates the proliferation of intestinal stem cells (ISCs) as well as tissue repair and facilitates the attraction of innate immune cells [19, 27]. Further, IL-22 controls the composition of the microbiota by driving the expression of AMPs like Reg3β, Reg3γ and S100 proteins [30]. Of note, IL-17 A and IL-22 can also synergize to induce a stronger antimicrobial response or neutrophil recruitment [31, 47].

On the other hand, the microbiota can influence the cellular phenotype of intestinal T_H_17 cells. First, intestinal T_H_17 cells display a functional heterogenicity. Dependent on the local microenvironment, T_H_17 cells can assume, for instance, either an anti-inflammatory or a pathogenic fate, among other functional states. Generally, T_H_17 cells of an anti-inflammatory fate engage in controlling the mucosal immune response and maintain intestinal homeostasis. In contrast, T_H_17 cells that obtain robust effector functions are required to clear out enteric infections [11, 16, 48–50]. However, their dysregulation drives the onset and progression of chronic inflammation like IBD [7]. Second, intestinal T_H_17 cells can display features of cellular plasticity, representing one aspect of their functional heterogenicity. In response to the local cytokine milieu, T_H_17 cells transdifferentiate to other CD4^+^ T_H_ cell subset. Thereby they extinguish the expression of their signature cytokine IL-17 A and acquire the expression of another distinct cytokine profile. This process can give rise to T_H_1-like pro-inflammatory exT_H_17 cells as well as induce the conversion towards full regulatory T cells [14, 17, 51]. The contribution of the microbiota to both concepts, T cell plasticity and their overall functional heterogenicity, has been well established [52–54]. Still, a more detailed understanding of these interactions is necessary when attempting to develop more customized therapeutic approaches for patients with IBD.

The complex biological functions of T_H_17 cell-derived cytokines are reflected by their context-dependent roles in intestinal pathology: IL-17 A plays a protective role in colonic inflammation driven by the adoptive transfer of CD4^+^CD45RB^high^CD25^−^ T cells into *Rag1*^−/−^ mice. In this model, the absence of T cell-derived IL-17 A led to exacerbated intestinal inflammation in recipient mice [55]. In contrast, a pathogenic role for IL-17 A has been reported in the absence of IL-10. Treatment with recombinant IL-23 led to the induction of IL-17 A, which was associated with increased intestinal pathology compared to untreated mice. This effect was attributed especially to IL-17 A downstream signaling that promotes the expression of pro-inflammatory mediators like TNFα and IL-6 [56].

IL-22 is upregulated both locally in the inflammatory lesions of the colon, and systemically in plasma samples from IBD-patients [57]. This increased expression reflects on IL-22 generally thought to possess a protective role in acute intestinal inflammation. However, when uncontrolled, the same properties defining the protective features of IL-22 in acute intestinal inflammation contribute in a long-term perspective to the pathogenesis of colorectal cancer (CRC) [28]. Thus, the expression of IL-22 at the mucosal interface must be tightly regulated. In part, this is mediated by a soluble receptor, the IL-22 binding protein (IL-22 bp). This endogenous inhibitor of IL-22 activity possesses a higher affinity to bind IL-22 than its membrane bound counterpart [58, 59]. I*n vivo* studies have described a dichotomous character for IL-22 intestinal pathology. IL-22 plays a protective role in dextran sulfate sodium (DSS) induced colitis, as well as in intestinal inflammation following adoptive transfer of CD4^+^CD45RB^high^CD25^−^ T cells, highlighting its antimicrobial capacities and its function to promote epithelial regeneration and wound healing [60]. In addition, IL-22 mediates protection in the spontaneous UC-like development of colitis in TCRαKO mice via the induction of mucin-related genes [61]. Mice harboring the intestinal flora of *Il22bp*^*−/−*^ mice were protected against intestinal infection with *Clostridium difficile*, highlighting the role of IL-22 to prevent dysbiosis [44, 45, 62]. In contrast, IL-22 plays a pathogenic role in spontaneous colitis development in *Il10*^*−/−*^ mice, colitis induced by an anti-CD40 antibody and exacerbates colitis caused by the adoptive transfer of CD4^+^CD45RB^low^CD25^−^ T cells [63–65]. Upon initial cellular stress, IL-22 together with interferon-α (IFN-α) synergizes to induce ileal inflammation [66]. Thus, the function of IL-22 is highly dependent on the inflammatory context, as it is protective in a rather T_H_17-like microenvironment and is detrimental in a T_H_1-like context. Recently, it has been proposed that differential activation of downstream IL-22R signaling distinguishes between beneficial and pathological effects in acute colitis. Specifically, STAT3 activation is linked to protective pathways, such as promoting mucus production, while STAT1 activation e.g. upon priming by type-I interferons (IFN) tends to induce pro-inflammatory mediators [67].

## Interactions of T_H_17 cells with commensal bacteria

Intestinal commensal microorganisms affect the T_H_17 immune response across different stages of their functional development. Different CD4^+^ T_H_ cell subsets emerge in response to specific luminal antigens that require certain effector functions [68, 69].

The microbiota, as well as its metabolic byproducts actively take part in driving the induction and differentiation of T_H_17 cells and regulate their cytokine production. Mice housed under germ-free (GF) conditions exhibit a substantially reduced population of T_H_17 cells, accompanied by an impaired expression of the respective cytokines in the gut [70]. Selectively depleting specific groups of intestinal bacteria by using different antibiotic cocktails revealed that individual strains can influence the immunomodulatory functions of SI LP T_H_17 cells. In intestinal homeostasis segmented filamentous bacterial (SFB) drive the differentiation of T_H_17 cells to produce IL-17 A and IL-22, but not IFNγ [71]. Several studies supported the concept of SFB-specific intestinal T_H_17 cells, now widely recognized for their role to shape the mucosal immune response. As classical commensal microorganisms SFB are considered a non-invading bacterial strain, which closely adheres to the apical membrane of IECs [72]. SFB that fail to establish a sufficient attachment do not induce the expression of distinct antimicrobial genes, such as *Saa*, *Reγ3b*, and *Nos2.* These molecules facilitate the induction of a milieu, which favors the generation of T_H_17 cells [73, 74]. SFB-positive *H2-ab1*^*−/−*^ mice show a significant decrease of T_H_17 cells indicating that MHC-II-dependent antigen recognition plays a central role in the differentiation of intestinal T_H_17 cells [68]. Particularly, CD11c^+^ LP mononuclear phagocytes (MNPs) were identified to induce a robust MHC-II-dependent T_H_17 response specific to SFB. Among LP MNPs CD11b^+^CD103^−^CD64^+^ intestinal macrophages, which express CX_3_CR1 are indispensable for SFB-specific antigen T_H_17 cell induction in vivo [75]. These observations suggest that SFB interact with antigen presenting cells (APCs) although they do not compromise the intestinal barrier and that bare changes in the respective cytokine environment and produced metabolites are not adequate to induce a T_H_17 cell response towards SFB. SFB specific antigens are transported via endocytic vesicles (EV) in a dynamin-dependent manner within the IECs to induce T_H_17 cells in the underlying LP [76]. In addition, as for other intestinal bacteria microfold cells (M cells) located in the dome region of the Peyer’s Patches (PPs) capture antigens, which reach the APCs through transcytosis [77]. Of note, the MHC-II-dependent differentiation and proliferation of intestinal T_H_17 cells does not necessarily require organized lymphatic tissue. Instead, microbial antigen presentation can occur directly in the LP [72, 75]. It remains an open question whether various APC subsets, activated by distinct microorganisms, differentially affect the functional priming of T_H_17 cells. Intriguingly, though SFB possess highly host specific genetics, a distinguishable human-specific genome has not yet been identified [78]. Thus, also other commensals can favor an environment to induce and differentiate T_H_17 cells:

In GF mice, monocolonization with individual human commensal strains revealed that *Bifidobacterium adolescentis* (BA) strongly induces IL-17 A production by SI CD4^+^ T_H_ cells. Both SFB and BA closely associate with the IECs, facilitating T_H_17 cell induction [73, 79]. Similarly, *Parabacteroides goldsteinii*, a commensal bacterium of the mammalian gut that is particularly abundant during fiber-rich diet, expresses the outer membrane protein A (OmpA1), which selectively induces IL-22 production by neutrophils and intestinal ILC3s, independent of secreted metabolites [80].

In addition to direct interactions, associated metabolic byproducts of commensal bacteria can influence intestinal T_H_17 differentiations. In vitro treatment of naïve CD4^+^ T_H_ cells with microbial metabolites such as butyrate, tryptophan derivatives and sphingolipids upregulate the transcription factors AhR and HIF1α, which in turn regulate IL-17 A and IL-22 expression [81, 82]. Furthermore, bile acids like 3-OxoLCA inhibit T_H_17 cell differentiation via direct binding to RORγt, while other compounds including lipoteichoic acid (LTA), vitamin B_6_ derivatives, and hydrogen sulfide (H_2_S) modulate cytokines that promote T_H_17 cell differentiation [83]. T_H_17 responses are also indirectly shaped through bacterial byproducts acting on APCs. Examples include polysaccharide A from *Bacteroides fragilis* and commensal-derived ATP, which interacts with P2X and P2Y receptors present on intestinal CD70^high^CD11c^low^ cells, likely corresponding to previously described CD11b^+^CD103^−^CD64^+^ MNPs. Stimulation of these receptors subsequently induces the production of IL-6 and TGF-β, key cytokines for T_H_17 cell lineage commitment [75, 84, 85]. Beyond direct interactions and metabolite-mediated effects, commensal microorganisms can drive T_H_17 responses via IgG-dependent mechanisms. In UC patients, certain commensal species trigger the production of anti-commensal IgG, forming immune complexes that bind the FCγR on intestinal macrophages. This activation induces IL-1β secretion, which in turn promotes T_H_17 differentiation and activation, linking commensal recognition to adaptive immune responses in an inflammatory context [86].

## Interactions of T_H_17 cells with pathobionts and pathogens

Disease-associated pathobionts and infectious pathogens also possess the ability to induce the differentiation and proliferation of intestinal T_H_17 cells (Table 2). GF mice exposed to the microbiota of patients with IBD exhibit increased frequencies of RORγt^+^ cells among CD4^+^ T_H_ cells, whereas the frequencies of Foxp3^+^ cells stay comparable. Furthermore, *Rag1*^*−/−*^ mice harboring microbiota from healthy controls are less susceptible to develop severe colitis following adoptive transfer of CD4^+^CD45RB^high^CD25^−^ T_H_ cells than their counterparts reconstituted with stool from patients with IBD [87]. Importantly, the relationship between these potentially harmful microorganisms and intestinal T_H_17 cells is bidirectional, since T_H_17 cell-derived cytokines contribute to multiple host defense mechanisms [24, 88]. Several specific microorganisms have been identified to promote T_H_17 response in the intestine through distinct mechanisms: *Actinobacterium Eggerthella lenta*, a pathobiont linked to several autoimmune disorders and highly enriched in IBD stool samples, is sufficient to activate intestinal IL-17 A^+^ CD4^+^ T_H_ cells. This activation is mediated via the cardiac glycoside reductase 2 (Cgr2), which metabolizes inhibitory substances of T_H_17 activity, thereby relieving suppression of RORγt [89]. Another pathobiont, *Helicobacter hepaticus*, can alter the cytokine profile of intestinal T_H_17 cells. In the absence of IL-10, *H. hepaticus* can drive the conversion of these cells into ex-T_H_17 cells in the gut. These cells cease the expression of IL-17 A and, passing through an IL-17 A^+^ INFγ^+^ DP state, eventually acquire exclusive production of INFγ [52, 90]. Yet, the impact of the microbiota on T_H_17 cell plasticity is still being explored. Although primarily studied in the context of CRC, *Enterotoxigenic Bacteroides fragilis* (ETBF), another pathogenic strain, can modulate the activity of T_H_17 cells. ETBF enhances CRC cell proliferation and promotes T_H_17 differentiation by downregulating the release of exosomal particles containing miR-149-3p, which normally suppresses T_H_17 cell activity. Overexpression of miR-149-3p in CD4^+^ T_H_ cells inversely correlates with the expression of IL-17 A, TNFα and RORγt [91].

In contrast to persistent pathobionts, acute infections by intestinal pathogens provide a distinct context for T_H_17 cell activation. During infection with *Salmonella Typhimurium*, bacterial amyloid fibrils specifically trigger the production of IL-17 A and IL-22 within 48 h. Interestingly, OmpA1, previously described in commensal bacteria, is also present on *S. Typhimurium* [80, 92]. In *Toxoplasma gondii* infection IL-22 and IL-17 A show an inverted expression pattern, whereas infection with *Citrobacter rodentium* leads to an upregulation of IL-17 A and IL-22 [30, 93]. However, only IL-22 is indispensable to clear out the infection.

### Interactions of T_H_17 cells with intestinal fungi

Fungi constitute an integral component of the intestinal microbiota. Recent studies underscore their involvement in the pathogenesis of chronic inflammatory disorders, including IBD, particularly under dysbiotic conditions [94]. Notably, elevated levels of *Candida albicans*,* Candida parapsilosis* and *Malassezia* have been reported in patients with CD [95]. Their ability to colonize the intestinal mucosa, rather than merely residing in the lumen of the GI-tract, contributes to their potent immunomodulatory functions [96]. Upon encountering fungal antigens, CX_3_CR1^+^ MNPs promote T_H_17 cell differentiation, similar to their role in sensing commensal bacteria [97]. In vitro, murine B cells have been shown to secrete IL-6 upon recognition of *Candida albicans* hyphae via TLR2, thereby promoting T_H_17 cell differentiation, collectively contributing to control luminal organisms [95, 98]. Studies in *wildling* mice, which harbor a complex microbiota including significantly more non-bacterial microorganisms, such as intestinal fungi, exhibit elevated baseline IL-17 A levels compared to conventional laboratory mice [99]. Especially mucosa-associated fungi have recently been recognized to increase the frequencies of RORγt^+^ T_H_17 cells in the colonic LP. Subsequently, the resulting higher levels of IL-17 A and IL-22 strengthens intestinal barrier integrity, thereby protecting the host from *C. rodentium* infection [96]. Intriguingly, colonization with *Candida albicans* elicits a T_H_17 response locally in the colon and extends this effect to systemic immunity, thereby enhancing neutrophil-mediated protection against disseminated infection [100, 101]. *Candida albicans* has emerged as a key driver of IL-17 A response in the intestine. Interestingly, some T_H_17 cells generated against other fungi, such as *Aspergillus fumigatus*, can also recognize *Candida albicans* antigens, suggesting a shared or cross-reactive antifungal T_H_17 repertoire [101]. Fungi can also influence the T_H_17 response by modulating the production of metabolites. During *Aspergillus* exposure, tryptophan metabolism, via the indoleamine 2,3-dioxygenase (IDO) in DCs favors the development of T_reg_ cells contributing to enhanced host defense while potentially allowing fungal persistence [102]. Nonetheless, this concept of fungal primed cross-reactive T_H_17 cells may also become pathogenic in the context of genetic susceptibility or chronic inflammatory conditions like IBD [103].

**Table 2:** Different components of the intestinal microbiota interact with TH17 cells

Intestinal Microbiota	Microorganism	Mechanism of action	Effect	Reference	
Commensal	Segmented filamentous bacteria	Close attachment and specific transcriptional program in IECs	Induces expression of IL-17 A and IL-22	[43, 73, 74]	
	*Bifidobacterium adolescentis*	Close attachment and specific transcriptional program in IECs	Induces expression of IL-17 A	[79]	
	*Parabacteroides goldsteinii*	Recognition of OmpA1	Induces expression of IL-22 by innate immune cells	[80]	
	Mucosa-associated fungi	Unknown	Induces expression of IL-17 A and IL-22 especially by adaptive immune cells	[96]	
	Different bacterial families	SCFA upregulate the expression of AhR and HIF1α	Induces expression of IL-17 A and IL-22	[81, 82]	
		ATP regulates cytokine production by CD70highCD11clow cells	Induces expression of IL-17 A	[84]	
		3-OxoLCA directly binds RORγt	Inhibits expression of IL-17 A	[83]	
Pathobiont	*Actinobacterium Eggerthella lenta*	Metabolism of RORγt inhibitors	Induces expression of IL-17 A and IL-17 F	[89]	
	*Helicobacter hepaticus*	Unknown	Induces transition to IFNγ^+^exT_H_17 cells	[90]	
	*Candida albicans*	Induces secretion of IL-6 by B cells	Induces expression of IL-17 A	[98]	
Pathogen	*Toxoplasma gondii*	Dependent on IL-23	Induces expression of IL-22 but not IL-17 A	[93]	
	*Citrobacter rodentium*	Dependent on IL-23	Induces expression of IL-22 especially by innate immune cells	[30]	
	*Enterotoxigenic Bacteroides fragilis*	Downregulation of miR-149-3p	Induces expression of IL-17 A	[91]	
	*Salmonella Typhimurium*	Amyloid fibrils induce TLR2/TLR1	Induces expression of IL-17 A and IL-22	[92]	

## Two of a same kind; T_H_17 cells of anti-inflammatory and pathogenic fate

**Fig. 1:**
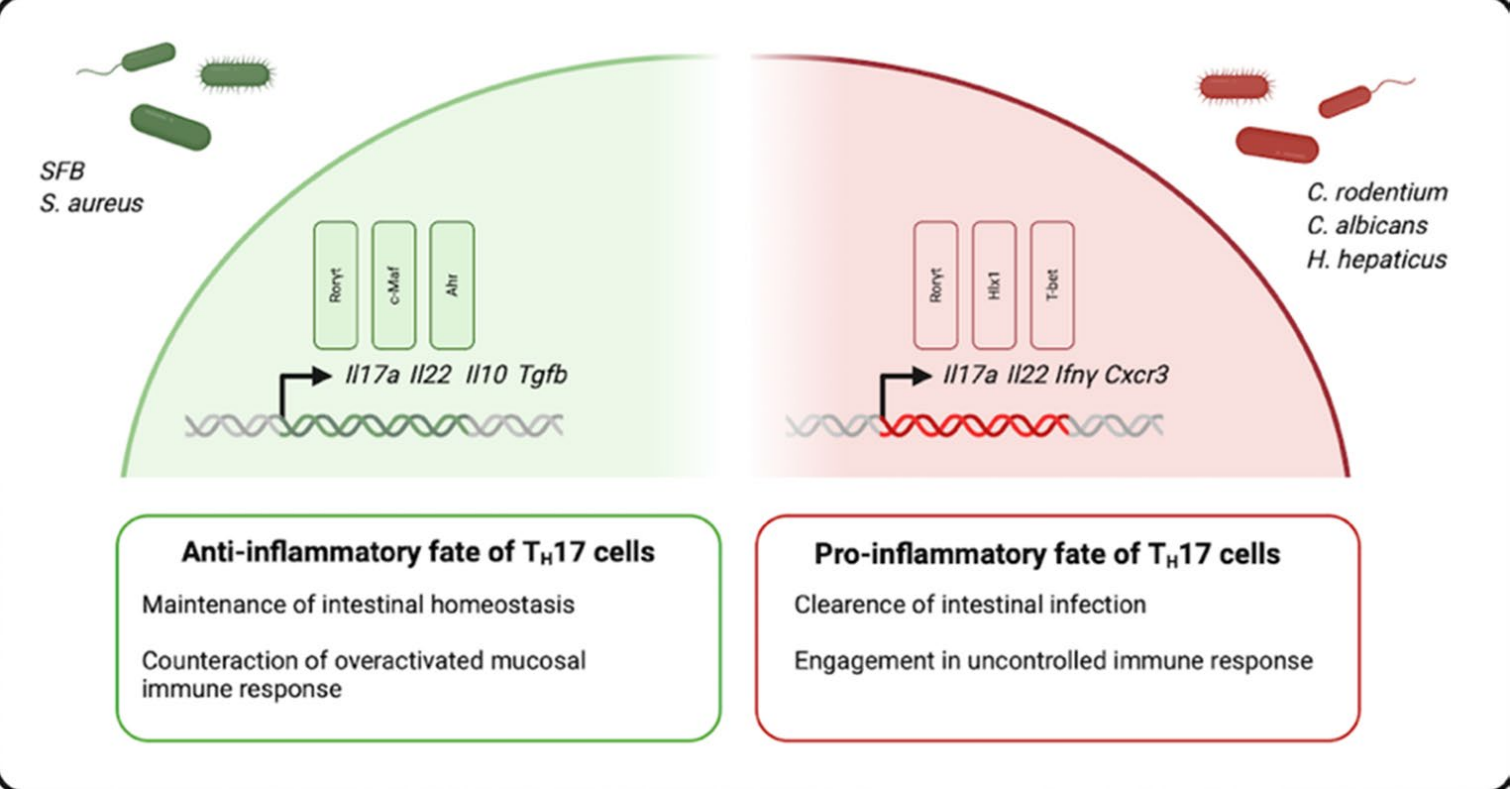
Distinct combinations of cytokines and components of the intestinal microbiota can shape the functional heterogeneity of T_H_17 cells. SFB and *S*. *aureus* are associated to drive the anti-inflammatory fate of T_H_17 cells, which express RORγt, c-Maf and Ahr. In contrast *C*. *rodentium*, *C*. *albicans* and *H*. *hepaticus* promote a pro-inflammatory phenotype of T_H_17 cells, which express RORγt, Hxl1 and Tbet. Created with BioRender.

T_H_17 cells functionally display a dichotomous character in the intestine. Although they consistently express IL-17 A, they can adopt either an anti-inflammatory or a pathogenic fate [7]. This dichotomy has been observed in both in vitro and in vivo studies [16, 49, 50]. T_H_17 cells of either fate can differentiate from a common naïve CD4^+^ precursor T_H_ cell [88, 104]. Differences in the surrounding cytokine milieu as well as specific components of the intestinal microbiota can influence their inflammatory properties (Fig. 1). 

T_H_17 cells with an anti-inflammatory fate are present under steady-state conditions, predominantly localized in the SI LP, and thus do not induce intestinal pathology [43]. They help maintain intestinal homeostasis by controlling luminal microorganisms, promoting epithelial barrier integrity, and preventing an overactivation of the mucosal immune response. The combination of IL-6, IL-1β and TGF-β1 drives the in vitro differentiation of murine T_H_17 cells toward an anti-inflammatory fate [16, 49]. In this context TGF-β1 plays a dual role, contributing to the differentiation of mature T_H_17 cells while also activating the transcription of Foxp3 in inducible T_reg_ (iT_reg_) cells [104]. Exposure of naïve CD4^+^ T_H_ cells to TGF-β1 alone drives iT_reg_ induction, whereas the additional presence of IL-6 or IL-1β shifts differentiation toward T_H_17 cells. Activation until this point is generally thought sufficient to maintain intestinal homeostasis. Further stages in the differentiation program are required to induce a substantial T_H_17 cell response to infection [105]. These anti-inflammatory properties are thought to be largely dependent on the polarization by TGF-β1. T_H_17 cells differentiated in vitro in the presence of a functional TGF-β1 signaling show an increased expression of immunoregulatory cytokines like IL-10 and an upregulation of inhibitory receptors [48, 53, 106]. In vivo, T_H_17 cells that differentiate in the presence of TGF-β1 and IL-6, but in the absence of IL-23, adopt a non-inflammatory phenotype, underscoring the context-dependent heterogeneity of T_H_17 cells [16]. Under homeostatic conditions, IL-10 production by intestinal macrophages restrains IL-23 signaling, which in an inflammatory context, would drive a pro-inflammatory T_H_17 response [107]. In humans, CD has been associated with reduced IL-10 production and elevated IL-23 secretion by dendritic cells (DCs), which might tip the balance toward a pro-inflammatory T_H_17 phenotype [108]. Intriguingly, in humans the classical combination of IL-6, IL-1β and TGF-β1 induces lower IL-17 A expression by naïve human CD4^+^ T_H_ cells after activation than IL-6 and IL-1β alone, whereas IL-23 has been identified as a potent driver of T_H_17 cell induction [109, 110]. In mice, SFB majorly contribute to induce T_H_17 cells of anti-inflammatory fate, which are characterized by the expression of coinhibitory receptors such as CTLA-4 and the secretion of IL-10, thereby restricting the proliferation of other naïve CD4^+^ T_H_ cells and contributing to intestinal immune homeostasis [15]. Naïve human CD4^+^ T_H_ cells cultured with *S. aureus* specific antigens were shown to differentiate into T_H_17 cells with regulatory properties, as they neither secrete INFγ nor express T-bet [54]. In contrast, T_H_17 cells with pathogenic capacities are implicated in both the onset and progression of intestinal pathology [7]. While they help to mount an efficient immune response against potential invading organisms, dysregulation of these cells can drive excessive, uncontrolled inflammation [16]. Moreover, they play a key role in the development of several autoimmune diseases [104]. Their unique transcriptional profile is further underscored by evidence that murine T_H_17 cells generated in vitro can differentiate from naïve CD4^+^ T_H_ cells even in the absence of TGF-β1. Notably, T_H_17 cells induced by the combination of IL-6, IL-1β and IL-23 exhibit a pronounced pro-inflammatory phenotype. They display accelerated proliferation and a distinct transcriptional signature characterized by the upregulation of the transcription factor T-bet, pro-inflammatory genes like *Cxcr3* and *Ccl9* alongside reduced expression of immunoregulatory molecules and coinhibitory receptors [7, 49, 50]. In addition, these cells are more prone to altering their cytokine profile. T_H_17 cells of anti-inflammatory fate predominantly produce IL-17 A and IL-22, whereas T_H_17 cells with proinflammatory capacities undergo plasticity, often adopting a T_H_1-like phenotype. To meet their increased energy demands, these cells shift toward glycolytic metabolism. In mice, colonic infections with *C. rodentium* triggers a T_H_17 immune response that shifts toward a T_H_1-like phenotype, accompanied by the production of INFγ [53]. Similarly, colonization with the pathobiont *Helicobacter hepaticus* induces iT_reg_ cells in homeostasis but drives a pro-inflammatory T_H_17 response in an immunocompromised host [36]. In humans, antigens derived from the pathobiont *Candida albicans* can induce simultaneous production of IL-17 A and INFγ, along with T-bet expression, which may promote further tissue damage [54]. 

## Conclusion and outlook for future translational aspects of T_H_17 cell-derived cytokines in IBD

Current therapies for IBD often involve broad modulation of the body’s inflammatory response, aiming to achieve steroid-free remission. CD4^+^ T_H_ cells are recognized as one of the major drivers of intestinal inflammation in both CD and UC, and elevated levels of T_H_17 cell-derived cytokines have been well documented in patients with IBD [57, 111–113]. Thus, one promising therapeutic approach has been thought to be the neutralization of T_H_17 cell-derived cytokines using specifically designed monoclonal antibodies. Targeting IL-17 A has been shown to be effective in certain chronic inflammatory conditions [114]. Hence, the therapeutic potential of the monoclonal antibodies Secukinumab and Ixekizumab, which selectively target IL-17 A, was assessed in patients with moderate to severe CD. Intriguingly, they failed to induce clinical remission, and clinical studies reported severe adverse effects in some cases [115, 116]. Efmarodocokin (UTTR1147A), a recombinant IL-22 agonist stabilized via fusion to a mutated crystallizable fragment (Fc) of human IgG4, was found to be safe in both healthy volunteers and patients with UC. This fusion protein binds the IL-22 receptor, activates downstream targets, and induces changes in the microbiota. These results reflect the complex role of IL-17 A and IL-22 in the inflamed colon, where they exert both protective and pathogenic effects, highlighting the need for future treatment strategies that preserve the beneficial functions T_H_17 cells in the intestine, while suppressing their inflammatory properties [62]. One example of this strategy is the use of selective IL-23 antibodies, including Guselkumab, Mirikizumab and Risankizumab, which target the p19 subunit of IL-23. Treatment with these biologicals suppresses pro-inflammatory T_H_17 cells while promoting the emergence of anti-inflammatory T cell subsets [117–119].

The intestinal microbiota plays a pivotal role in regulating T_H_17 function and their associated cytokines. Consequently, further research is needed to elucidate how the microbiota shapes the context-dependent roles of T_H_17 cell-derived cytokines. Such studies will provide a foundation for future strategies that either directly target the microbiota or use it as a biomarker to guide optimal therapeutic interventions.

## Data Availability

No original data was generated for this review article.

## Declarations

### Declarations

SH reports personal fees from AbbVie, Janssen, Lilly, Falk, BMS, and Ferring outside the submitted work.
